# A rare case of pyosalpinx in adolescent girl with Hirschsprung’s disease who underwent transvaginal ultrasound-guided drainage

**DOI:** 10.1186/s40792-023-01657-0

**Published:** 2023-05-09

**Authors:** Yu Sugai, Yoshiaki Kinoshita, Takashi Kobayashi, Yoshiaki Takahashi, Yuhki Arai, Toshiyuki Ohyama, Naoki Yokota, Shoichi Takano, Akiko Kobayashi

**Affiliations:** 1 Department of Pediatric Surgery, Niigata University Graduate School of Medical & Dental Sciences, 1-757 Asahimati-Dori, Chuo-Ku, Niigata, 951-8510 Japan; 2 Department of Obstetrics and Gynecology, Niigata University Medical and Dental Hospital, 1-754 Asahimati-Dori, Chuo-Ku, Niigata, 951-8520 Japan

**Keywords:** Pyosalpinx, Hydrosalpinx, Hirschsprung’s disease, Adolescent, Transvaginal drainage, Ultrasound guided drainage

## Abstract

**Background:**

Hydrosalpinx and pyosalpinx are rare gynecologic problems during adolescence, especially in girls without a history of sexual activity. They are even rarer in women with Hirschsprung’s disease (HD). We herein report a case of pyosalpinx in an adolescent girl with HD treated by transvaginal ultrasound-guided drainage.

**Case presentation:**

The present patient was a 12-year-old girl (weight 83 kg; height 159 cm; body mass index 32.8 kg/m^2^). She had undergone five laparotomies for long-segment HD by 2 years. Her menarche had occurred at 10 years. She was admitted with lower abdominal and anal pain. Computed tomography (CT), magnetic resonance imaging (MRI), and transvaginal ultrasound showed left pyosalpinx and abdominal abscess. Surgical drainage was necessary; however, she had a history of polysurgery and was severely obese, so laparotomy was considered to carry a high risk. Transvaginal ultrasound was deemed more likely to reach the abscess safely. Therefore, she was treated with transvaginal ultrasound-guided drainage by a gynecologist skilled in the procedure. She was discharged home after 52 days. One year and nine months after discharge, there was no reformation of either the abscess or pyosalpinx.

**Conclusions:**

Adolescent girls with HD are at risk of developing hydrosalpinx. Depending on the defecation status, pyosalpinx may also develop. As a less-invasive surgical treatment, transvaginal ultrasound-guided drainage can avoid laparotomy. Collaboration with a gynecologist is essential for the diagnosis and treatment of this clinical condition. Pediatric surgeons should communicate with gynecologists for such cases beginning around puberty for continuous follow-up.

## Background

Hirschsprung’s disease (HD) is a congenital disorder among neurocristopathies. HD is defined as the absence of ganglionic cells with hypertrophied cholinergic fibers in the myenteric and submucosal plexus of the distal rectum extending proximally for varying lengths, causing functional obstruction. HD remains a relatively rare congenital malformation [1], with a prevalence of approximately 1 in 5000 live births, and it has a high predisposition among males (male:female ratio approximately 3–4:1) in Japan [2, 3].

Hydrosalpinx is defined as fluid collection in the lumen of the fallopian tube owing to a distal obstruction. Menarche starts with a peak of stimulating hormones (FSH, LH), leading to an increase in glandular tissue and epithelial cells in the tubes. These changes subsequently activate the ovarian function and tubal motility. These events may lead to hydrosalpinx. Primary hydrosalpinx occurs because of inherent alterations in anatomy and physiology. However, secondarily acquired cases are the most frequent and include infections, adhesions, endometriosis, and neoplasms [1]. When intra-abdominal or vaginal infection occurs in hydrosalpinx, an abscess accumulates in the fallopian tube, resulting in pyosalpinx. However, hydrosalpinx and pyosalpinx are rare gynecologic problems during adolescence, especially in girls without a history of sexual activity [4].

We herein report a case of pyosalpinx in an adolescent girl with HD treated by transvaginal ultrasound-guided drainage.

## Case presentation

The present patient was a 12-year-old girl (weight 83 kg; height 159 cm; body mass index 32.8 kg/m2). She had undergone five laparotomies for long-segment HD by 2 years. After these operations, complications of anal stenosis and fecal incontinence occurred. She was followed up for these complications, but she had refused to undergo periodic medical examinations for the most recent 4 years. Her menarche had occurred at 10 years. The last menstrual period was 3 weeks prior to presentation, and the patient’s menstrual cycle was normal.

**Fig. 1:**
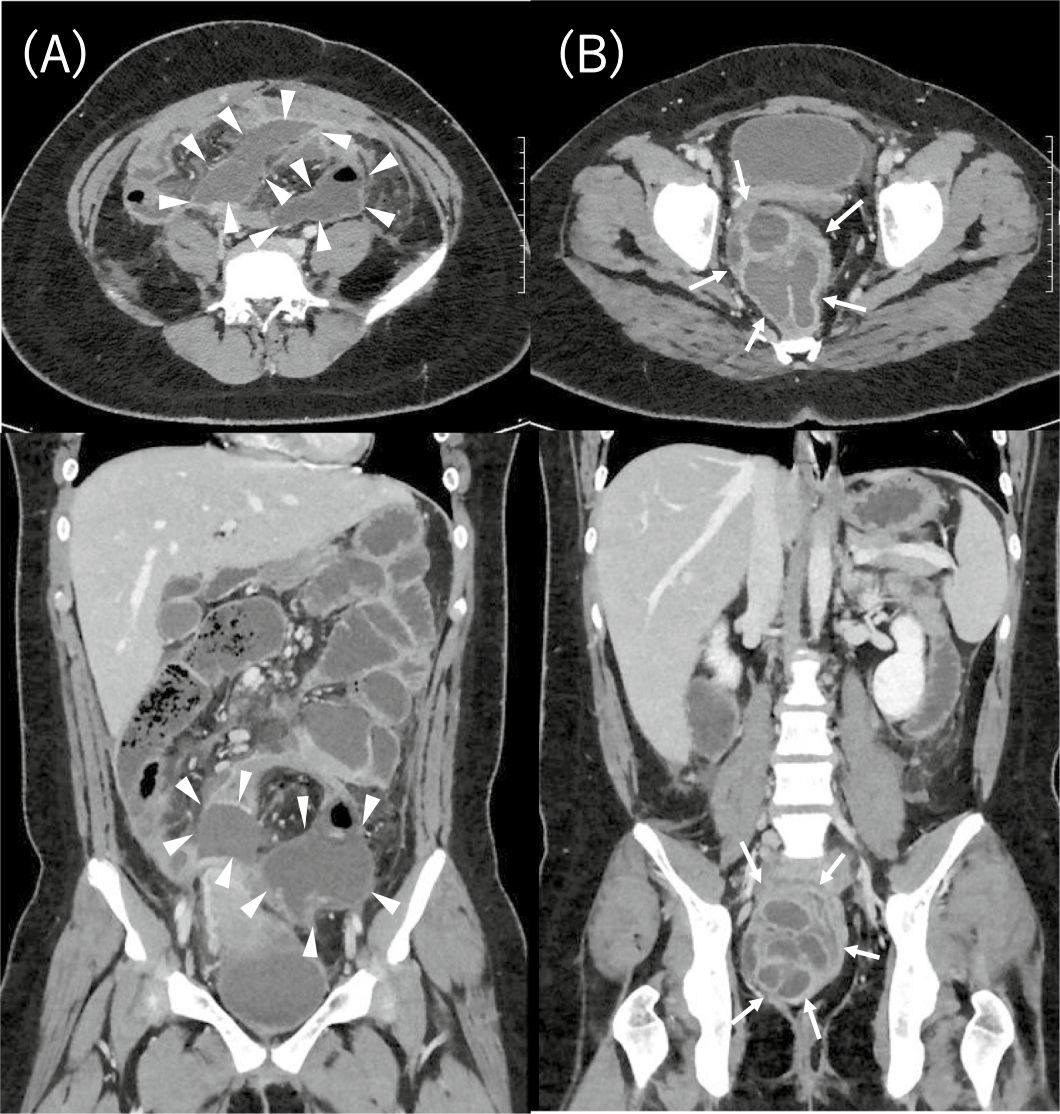
CT. **A** Abdominal abscess (white arrowhead). **B** Pyosalpinx (9 × 8 × 7 cm) (white arrow)

**Fig. 2:**
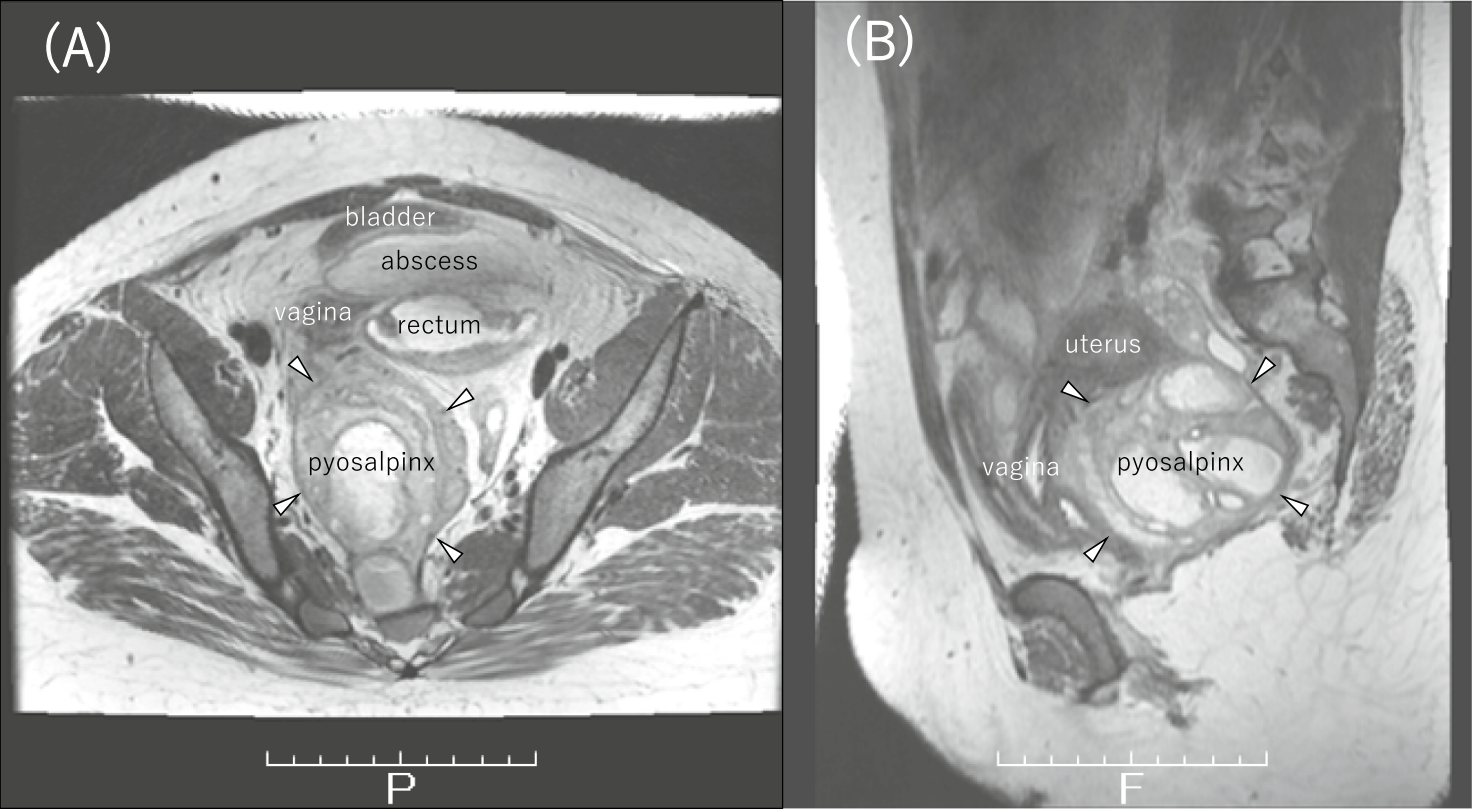
MRI. **A** Pyosalpinx is dorsal to the vagina and rectum (white arrowhead). The vagina is on the right side of the rectum and the abscess is between the bladder and rectum. **B** Pyosalpinx is dorsal to the vagina and uterus (white arrowhead)

She was admitted with lower abdominal and anal pain. Fever elevation (38.5 °C), tachycardia, and tachypnea were noted. Her underwear sometimes became soiled due to fecal incontinence; therefore, a pad was worn. Her laboratory tests revealed an inflammatory response and acute renal failure (WBC 21,250 µL, CRP 54.77 mg/dL, BUN 27 IU/L, Cre 1.21 mg/dL). Computed tomography (CT) showed a multicystic mass in the pelvis and abdominal cavity (Fig. 1). In addition, magnetic resonance imaging (MRI) demonstrated that the multicystic mass was dorsal to the vagina and rectum (Fig. 2). Transvaginal ultrasound showed findings compatible with those of CT and MRI. She was thus diagnosed with left pyosalpinx and abdominal abscess.

**Fig. 3:**
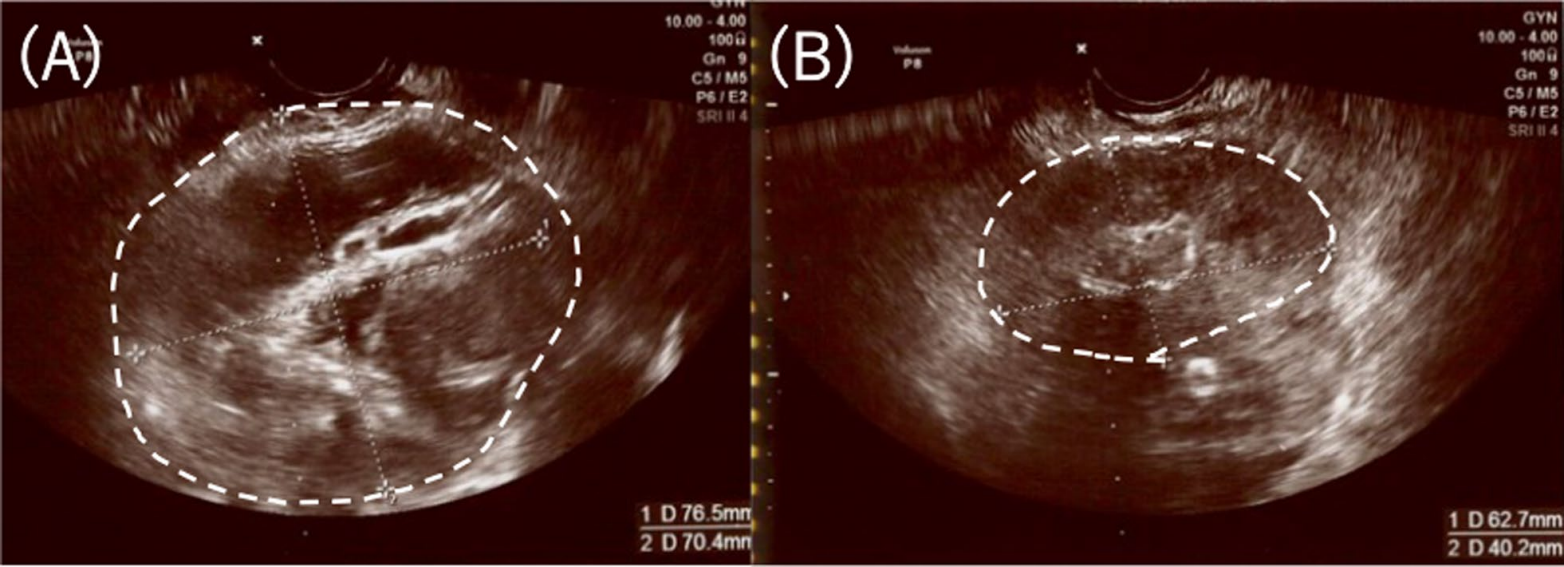
Transvaginal ultrasound-guided drainage for pyosalpinx. **A** Before drainage (7.6 × 7.0 cm). **B** After drainage (6.2 × 4.0 cm)

Surgical drainage was necessary, but she had a history of polysurgery and was severely obese, so laparotomy was considered to carry a high risk for her. Transvaginal ultrasound was deemed more likely to reach abscesses safely. Therefore, she was treated with transvaginal ultrasound-guided drainage by a gynecologist skilled in the procedure. Under transvaginal ultrasound, we used an 18G needle to obtain oocytes by puncturing into the pyosalpinx and abdominal abscess (Fig. 3). Drainage was performed five times during hospitalization. A total of 370 ml of purulent discharge was collected. Cultures from the pyosalpinx detected Bacteroides fragilis, Escherichia coli, and Fusobacterium nucleatum. Antibiotic therapy was also provided (TAZ/PIPC, MPEM, VCM, MCFG).

Furthermore, she had difficulty avoiding constant perineal fecal contamination because of her obesity. To improve the patient’s pubic hygiene, she underwent daily pubic washing and the insertion of chloramphenicol vaginal tablets. Once her activities of daily living had improved, the method of pubic washing was switched to self-cleaning, and she started wearing a menstrual cup all day to prevent retrograde vaginal infection. Enema and antidiarrheal drugs were used to improve fecal incontinence. Her general condition improved dramatically with multidisciplinary treatment.

**Fig. 4:**
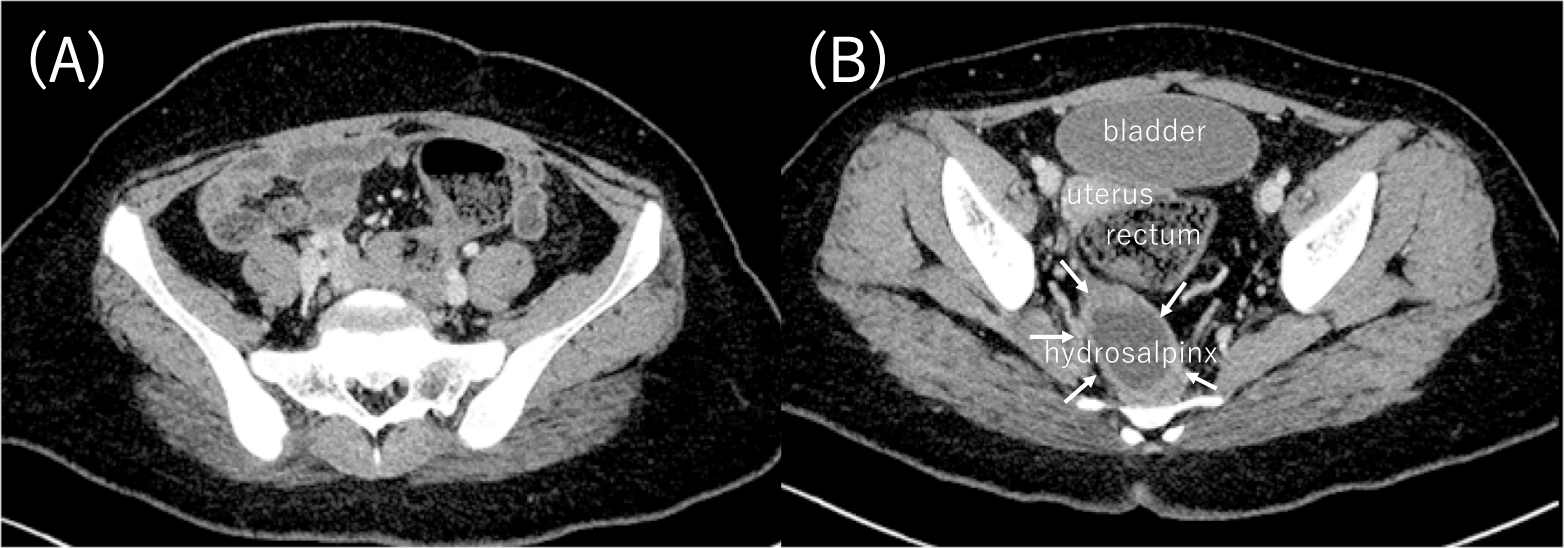
CT. **A** Abdominal abscess and pyosalpinx disappeared. **B** Hydrosalpinx was remained in the pelvis

She was discharged home after 52 days. CT was performed 1 month after discharge, and the abdominal abscess and pyosalpinx were found to have resolved (Fig. 4). The use of a menstrual cup was switched from all day to use only during the day. One year and nine months after discharge, the absence of abscess and pyosalpinx was confirmed. She is currently regularly seen by a pediatric surgeon and gynecologist.

## Discussion

Pyosalpinx in adolescent girls who are not sexually active is very rare and even rarer in women with HD [5]. According to a single-center study of HD, 17 cases with hydrosalpinx have been reported [1]. Concerning pyosalpinx, 22 patients were under 18 years, only one of whom reportedly had HD. Three of the 22 cases had poor defecation function [6]. To our knowledge, there have only been five reported cases of pyosalpinx with HD, including our own (Table 1). Most of these cases occurred after the onset of menstruation. Two patients had poor defecation control.

**Table1:** Overview of Pyosalpinx with HD

Years	Age (years)	Menarche (years)	Defecation function	Aganglionic bowel segment	HD Operation	Laparotomy (times)	Post operative complication	Treatment	Affected side
1997 [7]	11	11	Constipation	Short (Sigma)	Duhamel	2	Leak, intestinal obstruction	Salpingostomy	Left (right-hydrosalpinx)
2011 [8]	12	12	n.p	Long segment	TERPT	2	None	Antibiotic	Bilateral
2018 [1]	15	11	Unknown	TCA	Duhamel	3	Biopsy site perforation	Drainage(USD)	Bilateral
2019 [9]	11	Unknown	Unknown	Unknown	LA-Duhamel	2	Intestinal obstruction	Antibiotic	Right
2023 (Present case)	12	11	Soiling	Long segment	Swenson	5	Stoma falling	Drainage(USD)	Left

TCA: total colon aganglionosis; TERPT: transendrectal pull through; LA: laparoscopic assisted; USD: ultrasound guided drainage; n.p.: nothing particular

There have been several reports of an association between HD and hydrosalpinx. Pelvic surgery, pelvic inflammation, nerve damage, and neurological abnormalities have been noted. Furthermore, in patients with HD, smooth muscle cells in the fallopian tube or their innervation may be impaired and affect tubal peristalsis [10–13]. Recently, 5 of 17 girls (approximately 30%) who underwent surgery for HD were found to have hydrosalpinx, and all cases were bilateral, suggesting that congenital absence or reduction of fallopian tube nerve fibers may be involved [1]. In other words, not only acquired factors, such as surgery but also, the congenital factor of decreased nerve fibers related to HD may be a cause of hydrosalpinx. Therefore, it should always be kept in mind that HD girls may develop hydrosalpinx. Although this case is unilateral, considering the possibility of impaired fallopian tube function as well as adhesion, there is a possibility that contralateral hydrosalpinx may develop in the future, so careful follow-up is necessary.

Treatment of pyosalpinx includes conservative treatment (antibiotics), laparoscopic drainage, fluoroscopic drainage, laparoscopic salpingostomy, and fallopian tube resection [14]. Hospitalization for transvaginal echo-guided puncture drainage is reported to be shorter than that for antibiotic administration alone [15]. In the present case, transvaginal ultrasound-guided drainage was selected in cooperation with the gynecologist, and laparotomy was avoided. In HD patients, transabdominal drainage may be difficult because of intestinal adhesions due to a history of undergoing laparotomy several times. In addition, transrectal drainage may be difficult due to an abnormal pelvic anatomy, depending on the radical surgical approach applied. Therefore, a transvaginal approach is a useful route. In our case, the patient had both a history of polysurgery and obesity, so laparotomy or transabdominal ultrasound-guided drainage were considered high-risk procedures. Transvaginal drainage was very safe and effective in this case.

Fecal incontinence is a postoperative complication of HD. The present patient had undergone Swenson surgery and was in a state of chronic soiling. Poor defecation leads to poor pubic hygiene and increases the risk of developing the disease from retrograde infection of the vagina. Obesity leads to poor hygiene due to physical restraints [16]. In our case, thorough instruction in self-treatment and prevention of retrograde infection by wearing a menstrual cup were very effective in improving the vaginal environment.

Hydrosalpinx and pyosalpinx are common diseases in adult women but very rare in adolescent girls. Adolescent girls who have undergone HD surgery have a risk of developing hydrosalpinx. Furthermore, the risk of pyosalpinx depends on the defecation status. Therefore, pediatric surgeons should communicate with gynecologists beginning around puberty for continuous follow-up in these patients.

## Conclusion

Adolescent girls with HD are at risk of developing hydrosalpinx. Depending on the defecation status, pyosalpinx may develop. Defecation control is important for preventing pyosalpinx. As a less-invasive surgical treatment, transvaginal ultrasound-guided drainage can avoid laparotomy. Collaboration with a gynecologist is essential for making an accurate diagnosis and for performing appropriate treatment. Pediatric surgeons should communicate with gynecologists beginning around puberty for continuous follow-up of these patients.

## Data Availability

Not applicable.

## Abbreviations

HD: Hirschsprung’s disease
FSH: Follicle-stimulating hormone
LH: Luteinizing hormone
BMI: Body Mass Index
CRP: C-Reactive Protein
CT: Computed tomography
MRI: Magnetic Resonance Imaging
TAZ/PIPC: Tazobactam and piperacillin
MPEM: Meropenem
VCM: Vancomycin
MCFG: Micafungin
ADL: Active of Dairy Living
